# CircCode: A Powerful Tool for Identifying circRNA Coding Ability

**DOI:** 10.3389/fgene.2019.00981

**Published:** 2019-10-10

**Authors:** Peisen Sun, Guanglin Li

**Affiliations:** ^1^Key Laboratory of Ministry of Education for Medicinal Plant Resource and Natural Pharmaceutical Chemistry, Shaanxi Normal University, Xi’an, China; ^2^College of Life Sciences, Shaanxi Normal University, Xi’an, China

**Keywords:** bioinformatics, circular RNAs, ribosome profiling data, translation, coding potential, classification

## Abstract

Circular RNAs (circRNAs), which play vital roles in many regulatory pathways, are widespread in many species. Although many circRNAs have been discovered in plants and animals, the functions of these RNAs have not been fully investigated. In addition to the function of circRNAs as microRNA (miRNA) decoys, the translation potential of circRNAs is important for the study of their functions; yet, few tools are available to identify their translation potential. With the development of high-throughput sequencing technology and the emergence of ribosome profiling technology, it is possible to identify the coding ability of circRNAs with high sensitivity. To evaluate the coding ability of circRNAs, we first developed the CircCode tool and then used CircCode to investigate the translation potential of circRNAs from humans and *Arabidopsis thaliana*. Based on the ribosome profile databases downloaded from NCBI, we found 3,610 and 1,569 translated circRNAs in humans and *A. thaliana*, respectively. Finally, we tested the performance of CircCode and found a low false discovery rate and high sensitivity for identifying circRNA coding ability. CircCode, a Python 3–based framework for identifying the coding ability of circRNAs, is also a simple and powerful command line-based tool. To investigate the translation potential of circRNAs, the user can simply fill in the given configuration file and run the Python 3 scripts. The tool is freely available at https://github.com/PSSUN/CircCode.

## Introduction

Circular RNAs (circRNAs) are a special type of noncoding RNA molecule that has become a hot research topic in the field of RNA and is receiving a great deal of attention (Chen and Yang, 2015). Compared with traditional linear RNAs (containing 5′ and 3′ ends), circRNA molecules usually have a closed circular structure; rendering them more stable and less prone to degradation (Vicens and Westhof, 2014). Although the existence of circRNAs has been known for some time, these molecules were considered to be a by-product of RNA splicing. However, with the development of high-throughput sequencing and bioinformatics technologies, circRNAs have become widely recognized in animals and plants (Chen and Yang, 2015). Recent studies have also shown that a large number of circRNAs can be translated into small peptides in cells (Pamudurti et al., 2017) and have key roles despite their sometimes low level of expression (Hsu and Benfey, 2018; Yang et al., 2018). Although an increasing number of circRNAs are being identified, their functions in plants and animals generally remain to be studied. In addition to their functions as miRNA decoys, circRNAs have important translational potential, but no tools are available for specifically predicting the translational capabilities of these molecules (Jakobi and Dieterich, 2019).

Several tools do exist for the prediction and identification of circRNAs, such as CIRI (Gao et al., 2015), CIRCexplorer (Dong et al., 2019), CircPro (Meng et al., 2017), and circtools (Jakobi et al., 2018). Among them, CircPro can reveal translated circRNAs by calculating a translation potential score for circRNAs based on CPC (Kong et al., 2007), which is a tool for identifying the open reading frame (ORF) in a given sequence. However, because some circRNAs do not use the start codon during translation (Ingolia et al., 2011; Slavoff et al., 2013; Kearse and Wilusz, 2017; Spealman et al., 2018), employing CPC may filter out some truly translated circRNAs. In this study, we used BASiNET (Ito et al., 2018), which is an RNA classifier based on the machine learning methods (random forest and J48 model). It initially transforms the given coding RNAs (positive data) and noncoding RNAs (negative data) and represents them as complex networks; it then extracts the topological measures of these networks and constructs a feature vector to train the model that is used to classify the coding capacity of circRNAs. With this method, erroneous filtering of translated circRNAs that are not initiated by AUG is avoided. Additionally, Ribo-seq technology, which is based on high-throughput sequencing to monitor RPFs (ribosomal protected fragments) of transcripts (Guttman et al., 2013; Brar and Weissman, 2015), can be utilized to determine the locations of circRNAs that are being translated (Michel and Baranov, 2013). To identify the coding ability of circRNAs, we developed the tool CircCode, which involves a Python 3–based framework, and applied CircCode to investigate the translation potential of circRNAs from humans and *Arabidopsis thaliana*. Our work provides a rich resource for further study of the functions of circRNAs with coding capacity.

## Methods

**Figure 1:**
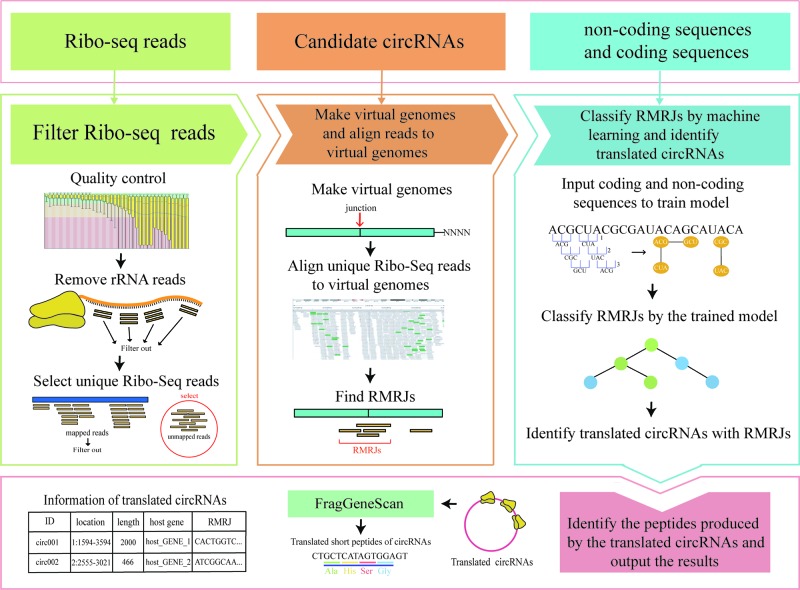
The workflow of CircCode. The top layer represents the input file required for each step of CircCode. The middle layer is divided into three parts, and each part represents a different stage of operation. From left to right, the first part represents the filtering of the Ribo-seq data; the quality control is executed by Trimmomatic, and the rRNA reads are removed by bowtie. The second part represents the steps used to produce the virtual genome and align the filtered reads to the virtual genome with STAR. The last part represents the identification of translated circRNAs by machine learning. The bottom layer represents the last step used to predict the peptides translated from the circRNAs and the final output results, including information on translated circRNAs and their translation products.

CircCode was written in the Python 3 programming language; it uses Trimmomatic (Bolger et al., 2014), bowtie (Langmead and Salzberg, 2012), and STAR (Dobin et al., 2013) to filter raw Ribo-seq reads and map these filtered reads to the genome. CircCode then identifies Ribo-seq read-mapped regions in circRNAs that contain junctions. After that, the candidate mapped sequences in the circRNAs are sorted based on classifiers (J48 model) into coding RNAs and noncoding RNAs by BASiNET. Finally, short peptides produced by translation are identified as potential coding regions of circRNAs. The entire process of CircCode consists of five steps (**Figure 1**).

### Filtering of Ribosomal Profiling Data

First, low-quality fragments and adapters in the Ribo-Seq reads are removed by Trimmomatic with the default parameters to obtain clean Ribo-seq reads. Second, these clean Ribo-seq reads are mapped to an rRNA library to remove reads derived from rRNA using bowtie. Because the read lengths of Ribo-seq are relatively short (generally less than 50 bp), it is possible for one read to match multiple regions. In this case, it is difficult to determine which region a particular read corresponds to. To avoid this, the clean Ribo-seq reads are mapped to the genome of a species of interest, and the reads that are not perfectly aligned to the genome are regarded as the final unique Ribo-seq reads.

### Assembling Virtual Genomes

CircRNAs usually appear as ring-shaped molecules in eukaryotes, and they can be identified based on their back-splicing junctions. However, the sequences of circRNAs in the fasta file are often in linear form. In theory, the result indicates that the junction is between the 5′ terminal nucleotide and the 3′ terminal nucleotide, although the junction and the sequence near the junction cannot be viewed directly, thus aligning Ribo-seq reads to circRNA sequences, including junctions, in a straightforward manner.

CircCode connects the sequence of each circRNA in tandem such that the junction for each is in the middle of the newly constructed sequence. We also separated each series unit by 100 N nucleotides to avoid confusion at the sequence alignment step (the length of each RPF is less than 50 bp). Finally, we obtained a virtual genome consisting only of candidate circRNAs in tandem separated by 100 Ns. Because CircCode focuses only on alignment between Ribo-seq reads and circRNA sequences, we can investigate the coding potential of circRNAs by mapping the Ribo-seq reads to this virtual genome, which can save a large amount of computational time (the virtual genome is much smaller than the whole genome) and increase the accuracy (by avoiding interference between upstream and downstream sequence comparisons of the circRNAs).

### Determination of the Ribo-seq Read-Mapped Region on a Junction (RMRJ) of circRNAs 

The final unique Ribo-seq reads are mapped to a previously created virtual genome using STAR. Because each tandem circRNA unit was separated by 100 N bases before producing the virtual genome, the largest intron length was set to not exceed 10 bases with the parameter “–alignIntronMax 10.” This parameter eliminates any interaction between different circRNAs in the sequence alignment. In the second step of virtual genome production, CircCode stores positional junction information for each circRNA in the virtual genome. If the Ribo-seq read-mapped region in the virtual genome includes the junction of the circRNA, and the number of mapped Ribo-seq reads on junction (NMJ) is greater than 3, the Ribo-seq reads-mapped region on junction of the circRNAs can be regarded as an RMRJ, which reveals a roughly translated segment of circRNAs near the junction site.

### Training of the Model and Classification of RMRJs

Although RMRJs can constitute powerful proof of translation, there are still some shortcomings in this method. Because the length of the reads of the ribosomal map is short, a read may be compared to the wrong position. Therefore, it is not convincing to simply consider the region covered by the Ribo-seq reads as the translated region. To this end, the machine learning method is used to identify the coding ability of the RMRJ. First, CircCode extracts coding RNAs (positive data) and noncoding RNAs (negative data) from a species of interest and uses them for model training by means of the difference in feature vectors between coding and noncoding RNAs. CircCode then uses the trained model to classify the RMRJs obtained in the previous step by BASiNET. If the RMRJ of a circRNA is recognized as coding RNA, then this circRNA can be identified as a translated circRNA.

### Prediction of Translated Peptides by RMRJs

As expression of circRNAs in organisms is low, Ribo-seq data do not show the exact 3-nt periodicity clearly in the case of fewer RPFs. Therefore, it is difficult to determine the exact translation start site of a translated circRNA. Due to the presence of a stop codon in some RMRJs and because the start codon is difficult to determine, the method of finding an ORF based on a start codon and a stop codon is not feasible.

To determine the true translation regions of these circRNAs and generate the final translation product, FragGeneScan (Rho et al., 2010), which can predict protein-coding regions in fragmented genes and genes with frameshifts, is used to determine the translated peptides produced by circRNAs.

To avoid the cumbersome running process, all the models can be called by a shell script; the user can simply fill in the given configuration file and input it into script, and the entire process for predicting the translated circRNAs will then be run. In addition, CircCode can be run separately, step by step, such that the user can adjust the parameters in the middle of the procedure and view the results of each step as desired.

## Results and Discussion

After testing on multiple computers, CircCode was found to run successfully with the required dependencies installed. To test the performance of CircCode, we used data for humans and *A. thaliana* to predict circRNAs with translation potential. The results were compared with circRNAs that have been verified experimentally as confirmation. Thereafter, we tested the false discovery rate (FDR) value of CircCode further. We used GenRGenS (Ponty et al., 2006) to generate a data set for testing based on known translated circRNAs and confirmed that the FDR value was within an acceptable range and at a low level. Finally, we evaluated the effect of different sequencing depths of Ribo-seq data on CircCode predictions and compared CircCode with other software.

### Translated circRNAs in Humans and *A. thaliana*

To apply the CircCode tool to real data, we first downloaded the files including the human reference genome GRCh38, genome annotation, and human rRNA, from Ensembl. For *A. thaliana*, the reference genomes (TAIR10), genome annotation files, and corresponding rRNA sequences were all downloaded from Ensembl Plants. The Ribo-seq data for humans and *A. thaliana* were downloaded from RPFdb (accession numbers: GSE96643, GSE81295, GSE88794) (Hsu et al., 2016; Willems et al., 2017), and all the candidate circRNAs from human and *A. thaliana* were downloaded from CIRCPedia v2 (Dong et al., 2018) and PlantcircBase, respectively (Chu et al., 2017). Ultimately, we identified 3,610 translated circRNAs from human and 1,569 translated circRNAs from *A. thaliana* using CircCode (**Supplementary Data 1**).

### Functional Enrichment of Human and *A. thaliana* circRNAs With Coding Potential

Using the CircCode results for human and *A. thaliana*, the online tool KOBAS 3.0 (Wu et al., 2006) was employed to annotate these translated circRNAs based on their parent genes. Furthermore, we performed GO (Gene Ontology) functional analysis and KEGG (Kyoto Encyclopedia of Genes and Genomes) enrichment analysis for these translated circRNAs using the R package clusterProfiler (Yu et al., 2012).

The KEGG results showed that the human circRNAs were enriched in protein processing in the endoplasmic reticulum pathway, carbon metabolism pathway, and RNA transport pathway. GO analysis indicated the participation of human translated circRNAs in the regulation of molecule binding, ATPase activity, and other RNA splicing-related biological processes. In addition, the translated circRNAs of *A. thaliana* are enriched in pathways related to stress resistance, suggesting that they play vital roles in this process (**Supplementary Data 2**).

### Accuracy Test for CircCode

To investigate the accuracy of CircCode, test sequences generated by GenRGenS, which uses the hidden Markov model to produce sequences that have the same sequence characteristics (such as the frequencies of different nucleotides, different codons and different nucleotides at the start of the sequence), were used.

For this study, we used previously published human translated circRNAs (Yang et al., 2017) as the input for GenRGenS and generated 10,000 sequences to test CircCode. We repeated the test 10 times, and on average, 27 translated circRNAs were predicted each time. The FDR value was calculated to be 0.0027, which is much less than 0.05, indicating that the predicted results are credible.

In addition, we compared the translated circRNAs from humans as identified by CircCode with verified polysome-associated circRNA data (Yang et al., 2017). Among them, 60% of the circRNAs were identified by CircCode (**Supplementary Data 3**).

### Influence of the Ribo-seq Data Sequencing Depth

**Figure 2:**
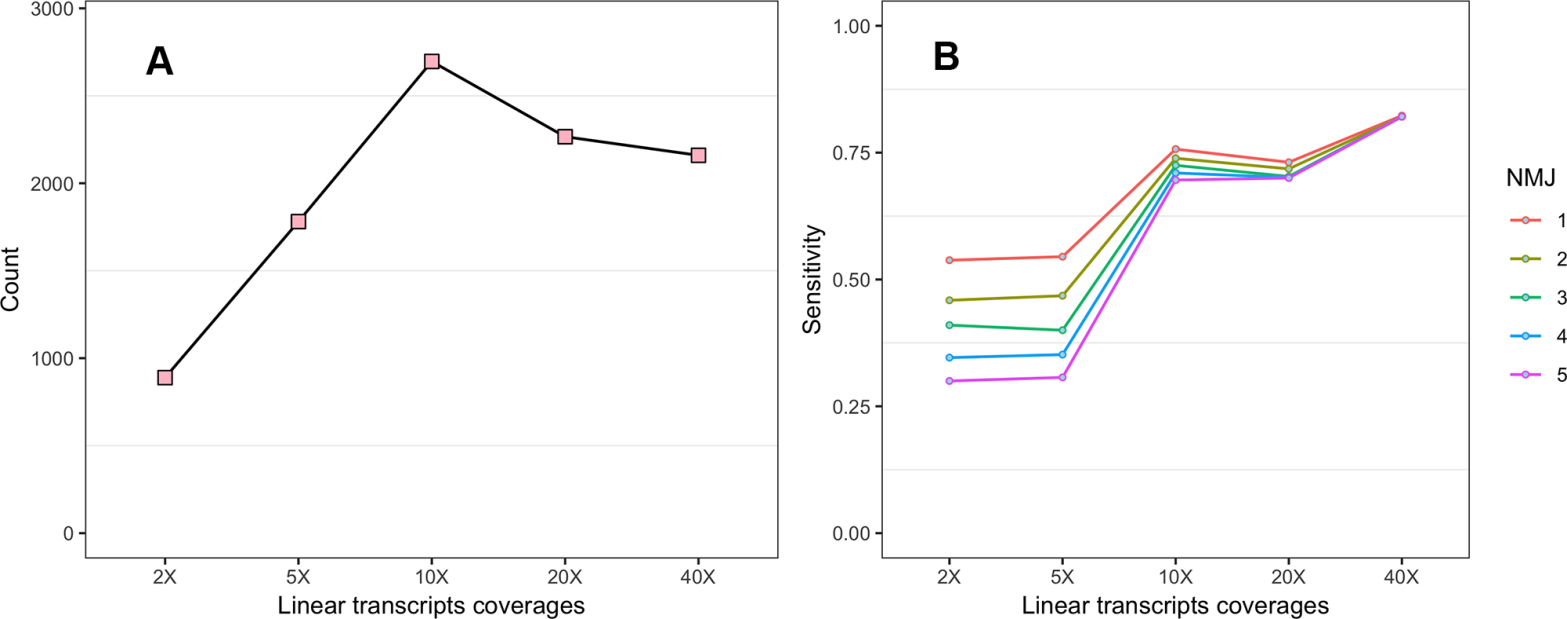
**(A)** Effect of Ribo-seq data sequencing depth on the predicted number of translated circRNAs. **(B)** The effect of junction read number (JRN) on CircCode sensitivity at different sequencing depths.

To investigate the impact of the sequencing depth of Ribo-seq data on the CircCode identification results, we first tested the effect of sequencing depth on the number of translated circRNAs (**Figure 2A**). When the sequencing depth was low, the predicted number of translated circRNAs was low, and the number of translated circRNAs increased with increasing sequencing depth. The number of translated circRNAs became stable when the sequencing depth reached no less than 10× linear transcript coverage.

Second, the influence of NMJ on sensitivity at different sequencing depths was also assessed (**Figure 2B**). The results showed that NMJ had less impact on sensitivity as the sequencing depth increased. CircCode also had higher sensitivity when using Ribo-seq data with higher sequencing depth.

### Comparison of CircCode With Other Tools

To compare CircCode with other tools, such as CircPro, the same set of Ribo-seq data (SRR3495999) from *A. thaliana* was used to identify translated circRNAs using six processors, with 16 gigabytes of RAM. CircPro identified 44 translated circRNAs in 13 min, whereas CircCode identified 76 translated circRNAs in 20 min. Thus, CircCode is more sensitive than CircPro at the same computer hardware level, but it takes more time. CircPro is concise and less time consuming than CircCode, but CircCode can identify more circRNAs with coding ability than CircPro.

## Conclusions

CircRNAs play an important role in biology, and it is crucial to accurately identify circRNAs with coding ability for subsequent research. Based on Python 3, we developed CircCode, an easy-to-use command line tool that has high sensitivity for identifying translated circRNAs from Ribo-Seq reads with high accuracy. CircCode exhibits good performance in both plants and animals. Future work will add the downstream character analysis to CircCode by visualizing each step in the process and optimizing the accuracy of the prediction.

## Availability and Requirements

CircCode is available at https://github.com/PSSUN/CircCode; operating system(s): Linux, programming languages: Python 3 and R; other requirements: bedtools (version 2.20.0 or later), bowtie, STAR, Python 3 packages (Biopython, Pandas, rpy2), R-packages (BASiNET, Biostrings). The installation packages for all of the required software are available on the CircCode homepage. Users do not need to download them individually. The CircCode home page also provides detailed user manuals for reference. The tool is freely available. There are no restrictions on use by nonacademics.

## Data Availability Statement

All relevant data are within the manuscript and its Supporting Information files.

## Author Contributions

Conceptualization: PS, GL. Data Curation: PS, GL. Formal Analysis: PS, GL. Writing – Original Draft: PS, GL. Writing – Review and Editing: PS, GL.

## Funding

This work was supported by grants from the National Natural Science Foundation of China (grant nos. 31770333, 31370329, and 11631012), the Program for New Century Excellent Talents in University (NCET-12-0896), and the Fundamental Research Funds for the Central Universities (no. GK201403004). The funding agencies had no role in the study, its design, the data collection and analysis, the decision to publish, or the preparation of the manuscript. The funders had no role in study design, data collection and analysis, decision to publish, or preparation of the manuscript.

## Conflict of Interest

The authors declare that the research was conducted in the absence of any commercial or financial relationships that could be construed as a potential conflict of interest.

## Supplementary Material

Supplementary Data 1 The sequence of the predicted translated circRNA and short peptide.

Supplementary Data 2 GO enrichment and KEGG enrichment results for humans and *Arabidopsis thaliana*.

Supplementary Data 3 Comparison of predicted translated circRNAs with validated translated circRNAs.
