# Supplementary material for: CircCode: A Powerful Tool for Identifying circRNA Coding Ability
Source: Front Genet. 2019 Oct 10;10:981. doi: 10.3389/fgene.2019.00981 (PMC6795751; doi:10.3389/fgene.2019.00981)
Supplement: Supplementary Data 2 — GO enrichment and KEGG enrichment results for humans and Arabidopsis thaliana. [file DataSheet_2.zip › Supplementary_data_2/human_GO.zip!/human_GO/HG_CC.pdf]

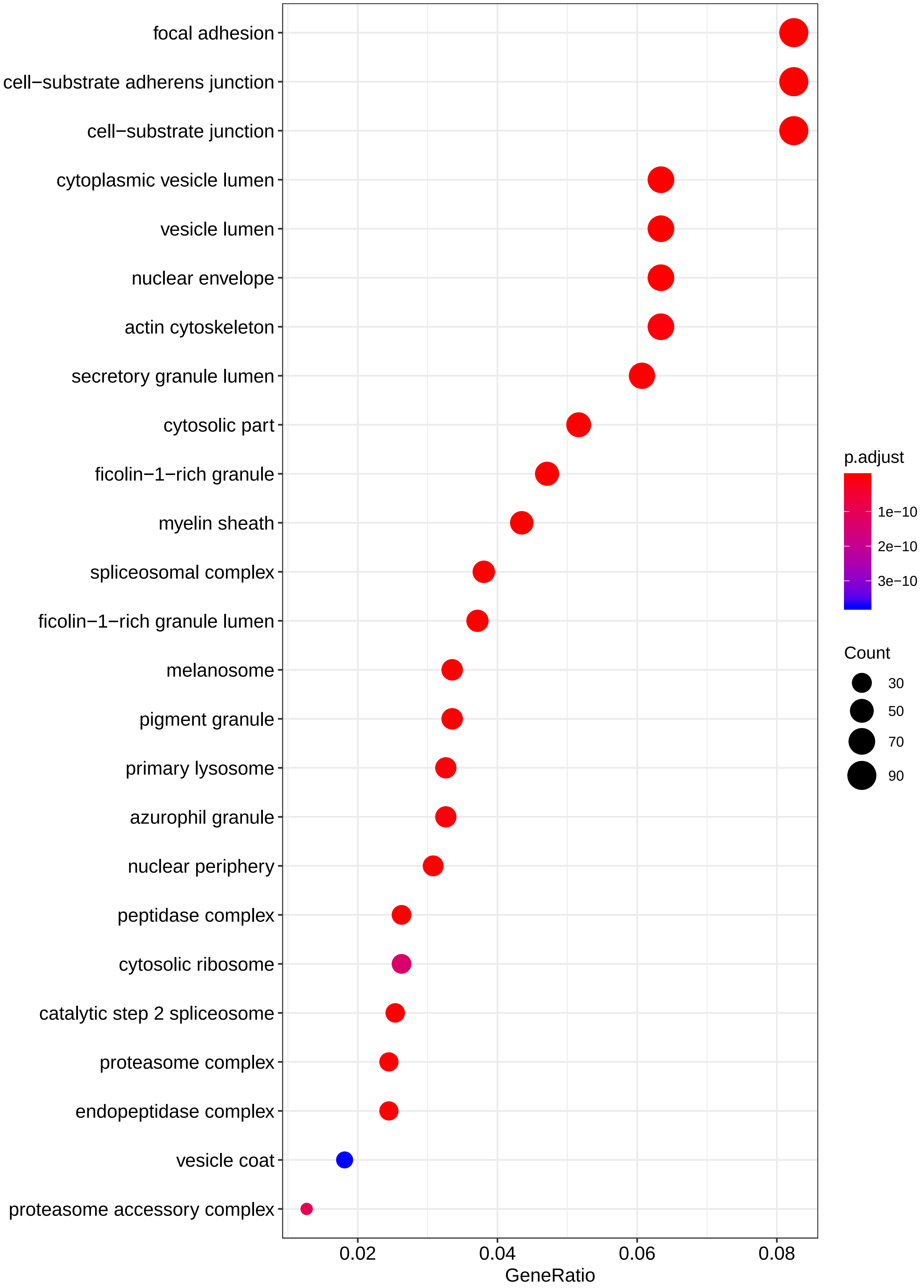

focal adhesion
cell−substrate adherens junction
cell−substrate junction
cytoplasmic vesicle lumen
vesicle lumen
nuclear envelope
actin cytoskeleton
secretory granule lumen
cytosolic part
ficolin−1−rich granule
myelin sheath
spliceosomal complex
ficolin−1−rich granule lumen
melanosome
pigment granule
primary lysosome
azurophil granule
nuclear periphery
peptidase complex
cytosolic ribosome
catalytic step 2 spliceosome
proteasome complex
endopeptidase complex
vesicle coat
proteasome accessory complex
0.02
0.04
0.06
0.08
GeneRatio
p.adjust
1e−10
2e−10
3e−10
Count
30
50
70
90
