# Supplementary material for: CircCode: A Powerful Tool for Identifying circRNA Coding Ability
Source: Front Genet. 2019 Oct 10;10:981. doi: 10.3389/fgene.2019.00981 (PMC6795751; doi:10.3389/fgene.2019.00981)
Supplement: Supplementary Data 2 — GO enrichment and KEGG enrichment results for humans and Arabidopsis thaliana. [file DataSheet_2.zip › Supplementary_data_2/human_GO.zip!/human_GO/HG_BP.pdf]

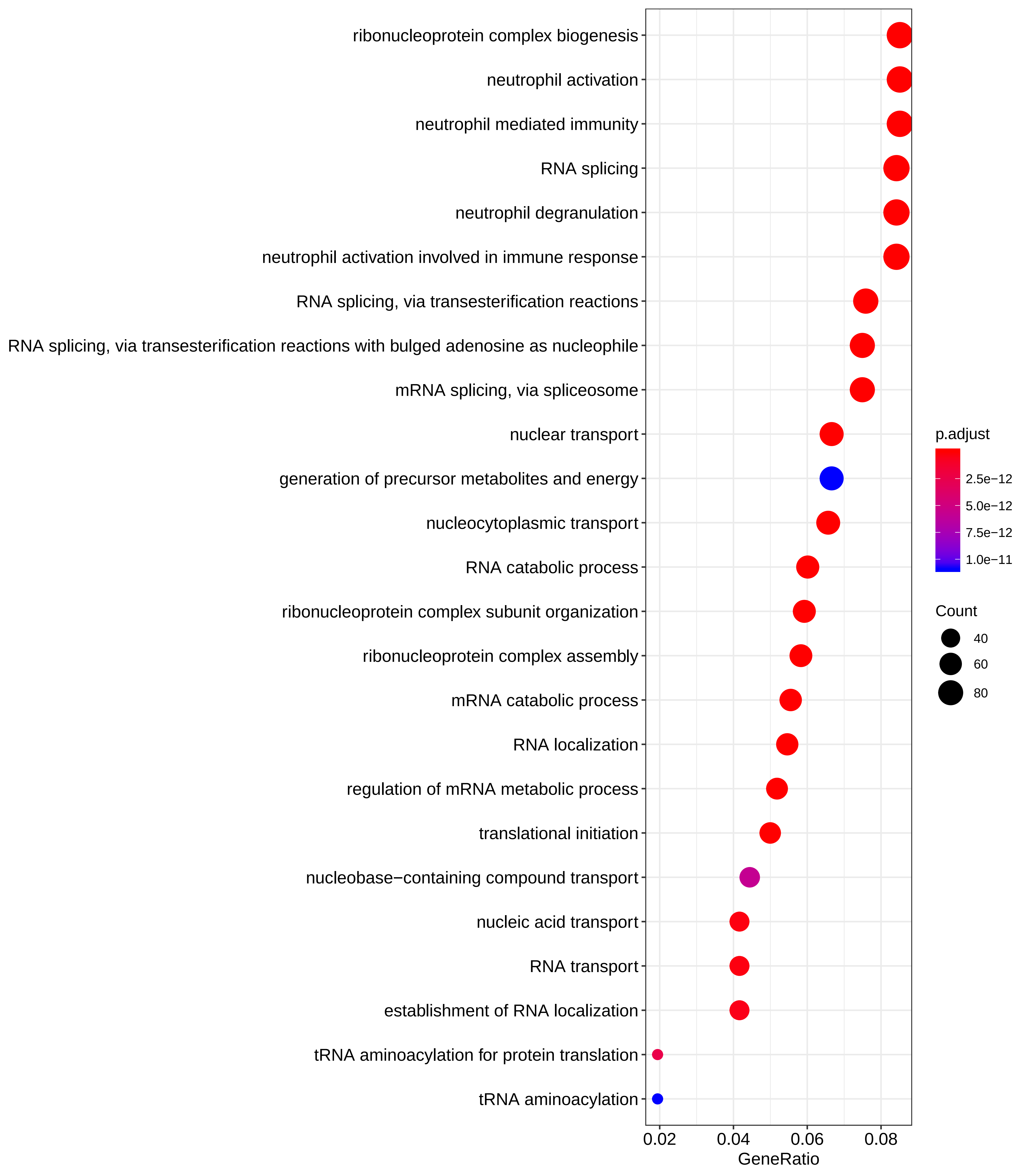
ribonucleoprotein complex biogenesis
neutrophil activation
neutrophil mediated immunity
RNA splicing
neutrophil degranulation
neutrophil activation involved in immune response
RNA splicing, via transesterification reactions
RNA splicing, via transesterification reactions with bulged adenosine as nucleophile
mRNA splicing, via spliceosome
nuclear transport
generation of precursor metabolites and energy
nucleocytoplasmic transport
RNA catabolic process
ribonucleoprotein complex subunit organization
ribonucleoprotein complex assembly
mRNA catabolic process
RNA localization
regulation of mRNA metabolic process
translational initiation
nucleobase−containing compound transport
nucleic acid transport
RNA transport
establishment of RNA localization
tRNA aminoacylation for protein translation
tRNA aminoacylation
0.02
0.04
0.06
0.08
GeneRatio
p.adjust
2.5e−12
5.0e−12
7.5e−12
1.0e−11
Count
40
60
80
