# Supplementary material for: CircCode: A Powerful Tool for Identifying circRNA Coding Ability
Source: Front Genet. 2019 Oct 10;10:981. doi: 10.3389/fgene.2019.00981 (PMC6795751; doi:10.3389/fgene.2019.00981)
Supplement: Supplementary Data 2 — GO enrichment and KEGG enrichment results for humans and Arabidopsis thaliana. [file DataSheet_2.zip › Supplementary_data_2/human_GO.zip!/human_GO/HG_MF.pdf]

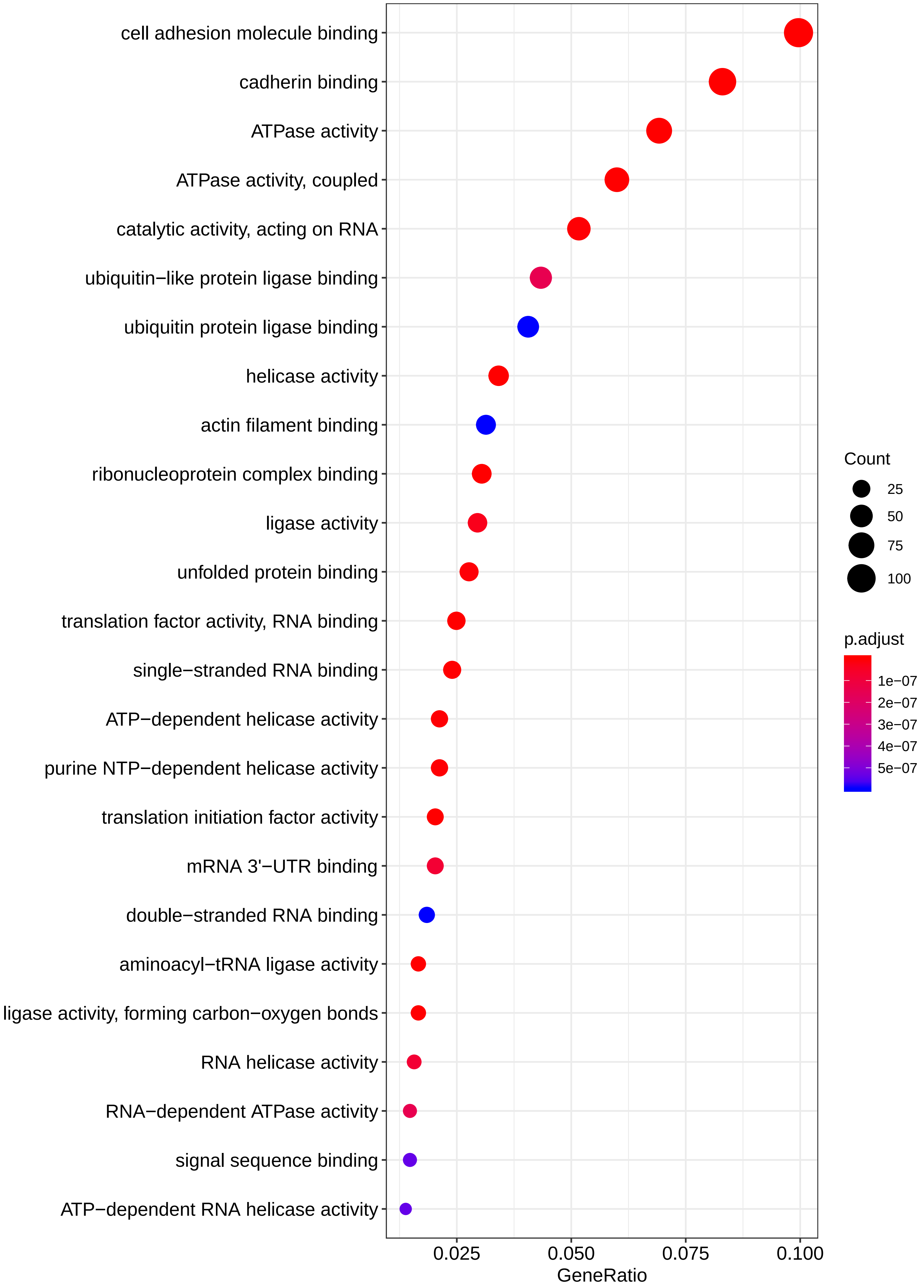

cell adhesion molecule binding
cadherin binding
ATPase activity
ATPase activity, coupled
catalytic activity, acting on RNA
ubiquitin−like protein ligase binding
ubiquitin protein ligase binding
helicase activity
actin filament binding
ribonucleoprotein complex binding
ligase activity
unfolded protein binding
translation factor activity, RNA binding
single−stranded RNA binding
ATP−dependent helicase activity
purine NTP−dependent helicase activity
translation initiation factor activity
mRNA 3'−UTR binding
double−stranded RNA binding
aminoacyl−tRNA ligase activity
ligase activity, forming carbon−oxygen bonds
RNA helicase activity
RNA−dependent ATPase activity
signal sequence binding
ATP−dependent RNA helicase activity
0.025
0.050
0.075
0.100
GeneRatio
Count
25
50
75
100
p.adjust
1e−07
2e−07
3e−07
4e−07
5e−07
