# Supplementary material for: CircCode: A Powerful Tool for Identifying circRNA Coding Ability
Source: Front Genet. 2019 Oct 10;10:981. doi: 10.3389/fgene.2019.00981 (PMC6795751; doi:10.3389/fgene.2019.00981)
Supplement: Supplementary Data 2 — GO enrichment and KEGG enrichment results for humans and Arabidopsis thaliana. [file DataSheet_2.zip › Supplementary_data_2/ath_GO.zip!/ath_GO/AT_MF.pdf]

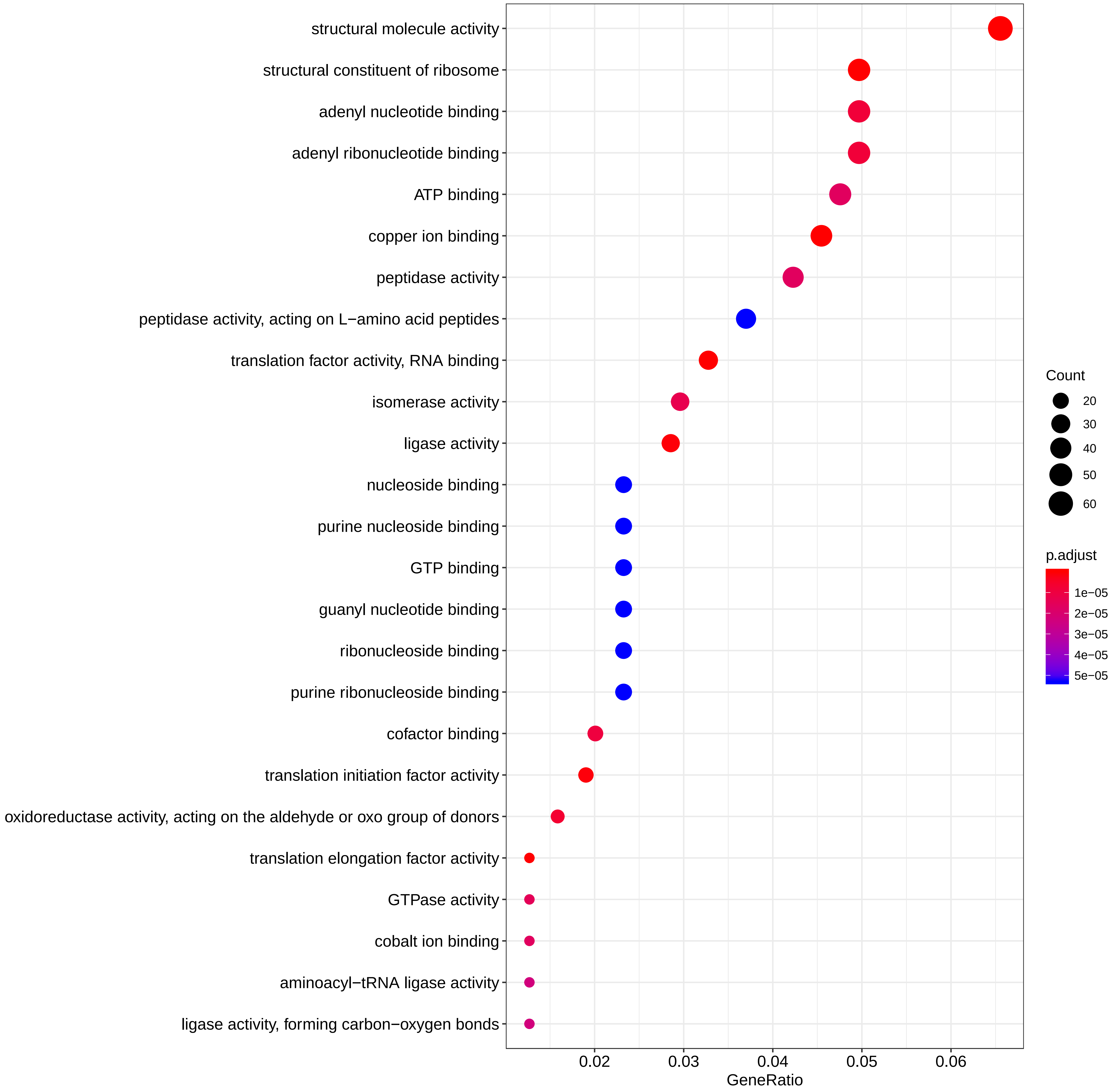
structural molecule activity
structural constituent of ribosome
adenyl nucleotide binding
adenyl ribonucleotide binding
ATP binding
copper ion binding
peptidase activity
peptidase activity, acting on L−amino acid peptides
translation factor activity, RNA binding
isomerase activity
ligase activity
nucleoside binding
purine nucleoside binding
GTP binding
guanyl nucleotide binding
ribonucleoside binding
purine ribonucleoside binding
cofactor binding
translation initiation factor activity
oxidoreductase activity, acting on the aldehyde or oxo group of donors
translation elongation factor activity
GTPase activity
cobalt ion binding
aminoacyl−tRNA ligase activity
ligase activity, forming carbon−oxygen bonds
0.02
0.03
0.04
0.05
0.06
GeneRatio
Count
20
30
40
50
60
p.adjust
1e−05
2e−05
3e−05
4e−05
5e−05
