# Supplementary material for: CircCode: A Powerful Tool for Identifying circRNA Coding Ability
Source: Front Genet. 2019 Oct 10;10:981. doi: 10.3389/fgene.2019.00981 (PMC6795751; doi:10.3389/fgene.2019.00981)
Supplement: Supplementary Data 2 — GO enrichment and KEGG enrichment results for humans and Arabidopsis thaliana. [file DataSheet_2.zip › Supplementary_data_2/ath_GO.zip!/ath_GO/AT_BP.pdf]

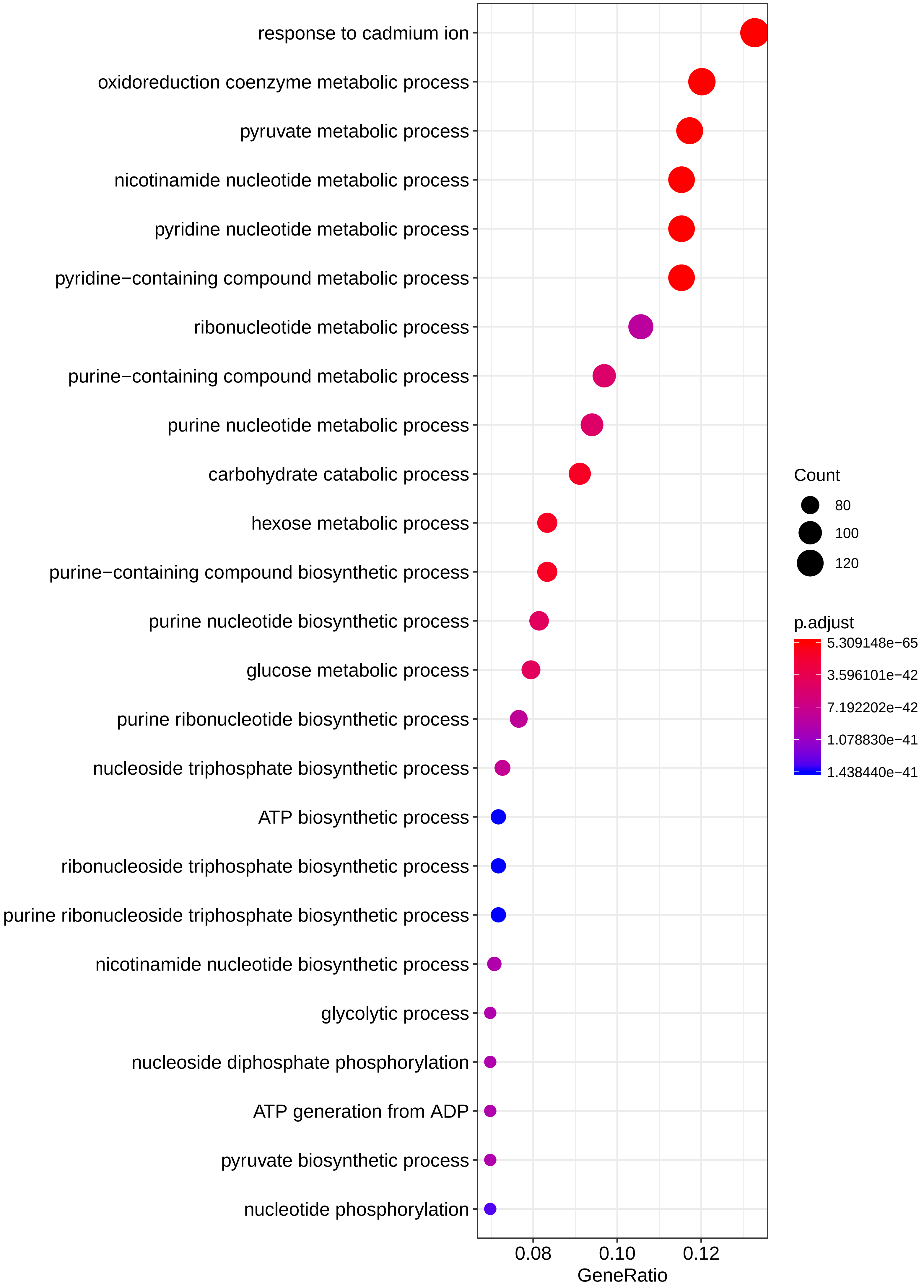

response to cadmium ion
oxidoreduction coenzyme metabolic process
pyruvate metabolic process
nicotinamide nucleotide metabolic process
pyridine nucleotide metabolic process
pyridine−containing compound metabolic process
ribonucleotide metabolic process
purine−containing compound metabolic process
purine nucleotide metabolic process
carbohydrate catabolic process
hexose metabolic process
purine−containing compound biosynthetic process
purine nucleotide biosynthetic process
glucose metabolic process
purine ribonucleotide biosynthetic process
nucleoside triphosphate biosynthetic process
ATP biosynthetic process
ribonucleoside triphosphate biosynthetic process
purine ribonucleoside triphosphate biosynthetic process
nicotinamide nucleotide biosynthetic process
glycolytic process
nucleoside diphosphate phosphorylation
ATP generation from ADP
pyruvate biosynthetic process
nucleotide phosphorylation
0.08
0.10
0.12
GeneRatio
Count
80
100
120
p.adjust
5.309148e−65
3.596101e−42
7.192202e−42
1.078830e−41
1.438440e−41
