# Supplementary material for: CircCode: A Powerful Tool for Identifying circRNA Coding Ability
Source: Front Genet. 2019 Oct 10;10:981. doi: 10.3389/fgene.2019.00981 (PMC6795751; doi:10.3389/fgene.2019.00981)
Supplement: Supplementary Data 2 — GO enrichment and KEGG enrichment results for humans and Arabidopsis thaliana. [file DataSheet_2.zip › Supplementary_data_2/ath_GO.zip!/ath_GO/AT_CC.pdf]

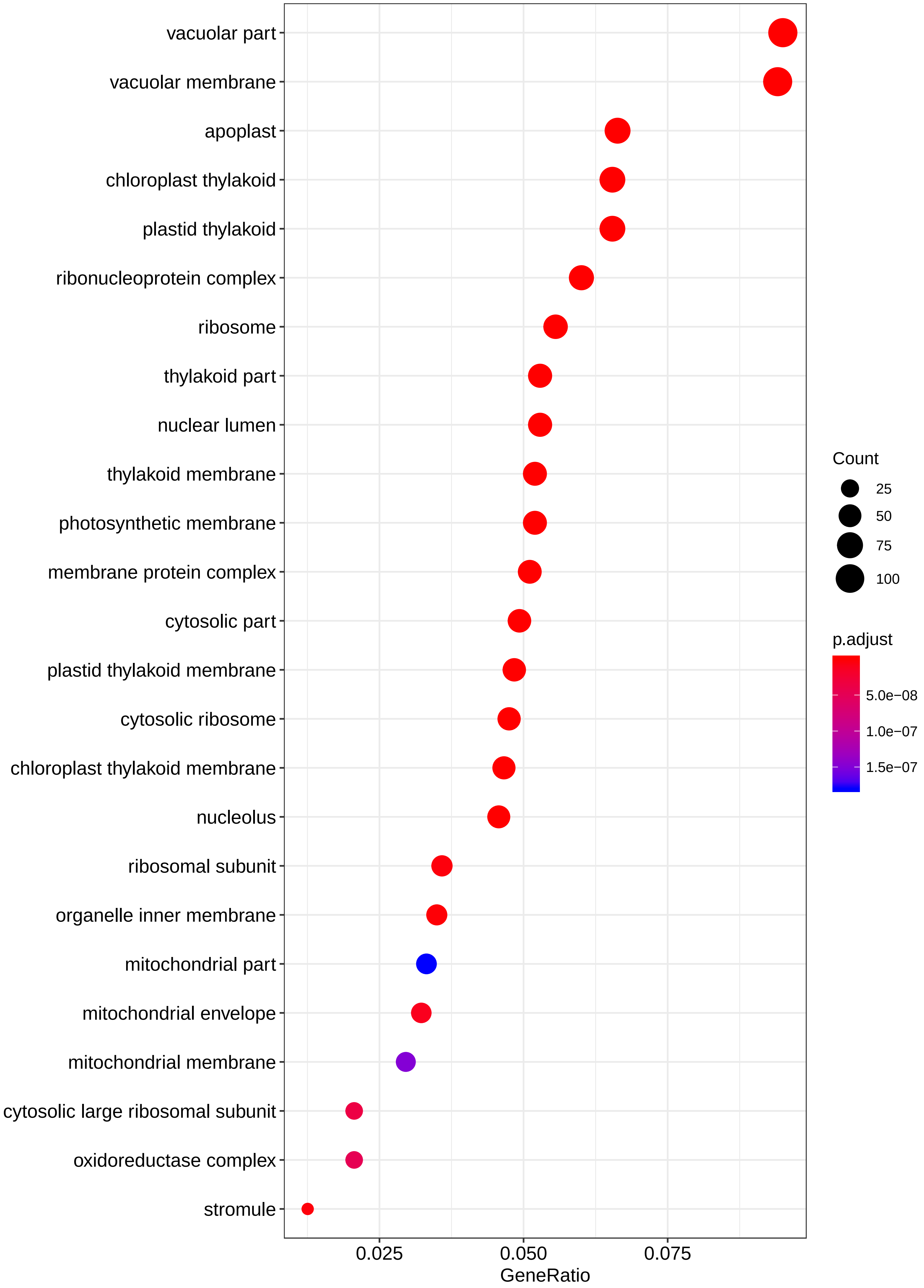
vacuolar part
vacuolar membrane
apoplast
chloroplast thylakoid
plastid thylakoid
ribonucleoprotein complex
ribosome
thylakoid part
nuclear lumen
thylakoid membrane
photosynthetic membrane
membrane protein complex
cytosolic part
plastid thylakoid membrane
cytosolic ribosome
chloroplast thylakoid membrane
nucleolus
ribosomal subunit
organelle inner membrane
mitochondrial part
mitochondrial envelope
mitochondrial membrane
cytosolic large ribosomal subunit
oxidoreductase complex
stromule
0.025
0.050
0.075
GeneRatio
Count
25
50
75
100
p.adjust
5.0e−08
1.0e−07
1.5e−07
